# Ideal for a Medical Society

**Published:** 1889-07

**Authors:** 


### Ideal for a Medical Society.

In his interesting Presidential address before the Medical Association of Central New York, on May 21, Dr. William C. Bailey, of Albion, N. Y., presented the following picture of what a medical society should be:

"A medical society, to serve its highest purpose, ought to be the standard-bearer of the profession; the magnet that will attract the physician from those secluded caverns of habit in which he, above all others, easily obscures himself—a lever that will lift the wheels of his professional chariot out of the ruts of established routine. It should be the physician's Mecca, whither he may journey, a pilgrim consecrating himself anew in his faith. It should be his Olympia, where he may seek social intercourse and friendship, a recreation for his wearied mind and body. It should be to him an Academia that will broaden his views of life, establish or strengthen religious principles, stimulate and elevate his moral nature. It should be his Athens, whither he may go for increased wisdom, learning in discussion, as did the philosophers of old, obtaining here a knowledge of all progress in operation, treatment, and literature. Finally, it may be his Delphi—the inspiration of his imagination; for in medicine, as in all science, it is often the prophetic finger of Fancy that points the way to the undiscovered. The world is made up of individuals. In the individual we find the unification of various qualities; and his success is assured who correlates—or, if I am permitted, educates—those qualities into such perfect harmony that they may attain the highest good. As each component part has its duties, the proper performance of which is essential, if the highest degree of perfection possible be desired, so in organized society the individual can not wholly escape certain obligations that rest upon him, even though like Byron's Manfred, he exile himself to the solitude of the Alps."

THE Board of Health of Philadelphia resolved, on June 18, to take legal measures to prevent the running of the passenger steamers on the Schuylkill river at Fairmount until the terms of the resolution of the Board relative to certain sanitary defects on the boats are carried out.

